# Detection of 22 common leukemic fusion genes using a single-step multiplex qRT-PCR-based assay

**DOI:** 10.1186/s13000-017-0634-3

**Published:** 2017-07-25

**Authors:** Xiaodong Lyu, Xianwei Wang, Lina Zhang, Zhenzhu Chen, Yu Zhao, Jieying Hu, Ruihua Fan, Yongping Song

**Affiliations:** 1 School of Basic Medical Sciences, Zhengzhou University, Zhengzhou, Henan 450000 China; 2 Central Laboratory, the Affiliated Cancer Hospital of Zhengzhou University; Henan Cancer Hospital, Zhengzhou, Henan 450000 China; 3 Department of Hematology, the Affiliated Cancer Hospital of Zhengzhou University; Henan Cancer Hospital, Zhengzhou, Henan 450000 China

**Keywords:** Leukemia, F-qRT-PCR, AML, MDS, CLL, ALL, Chromosomal rearrangement, Gene fusion

## Abstract

**Background:**

Fusion genes generated from chromosomal translocation play an important role in hematological malignancies. Detection of fusion genes currently employ use of either conventional RT-PCR methods or fluorescent in situ hybridization (FISH), where both methods involve tedious methodologies and require prior characterization of chromosomal translocation events as determined by cytogenetic analysis. In this study, we describe a real-time quantitative reverse transcription PCR (qRT-PCR)-based multi-fusion gene screening method with the capacity to detect 22 fusion genes commonly found in leukemia. This method does not require pre-characterization of gene translocation events, thereby facilitating immediate diagnosis and therapeutic management.

**Methods:**

We performed fluorescent qRT-PCR (F-qRT-PCR) using a commercially-available multi-fusion gene detection kit on a patient cohort of 345 individuals comprising 108 cases diagnosed with acute myeloid leukemia (AML) for initial evaluation; remaining patients within the cohort were assayed for confirmatory diagnosis. Results obtained by F-qRT-PCR were compared alongside patient analysis by cytogenetic characterization.

**Results:**

Gene translocations detected by F-qRT-PCR in AML cases were diagnosed in 69.4% of the patient cohort, which was comparatively similar to 68.5% as diagnosed by cytogenetic analysis, thereby demonstrating 99.1% concordance. Overall gene fusion was detected in 53.7% of the overall patient population by F-qRT-PCR, 52.9% by cytogenetic prediction in leukemia, and 9.1% in non-leukemia patients by both methods. The overall concordance rate was calculated to be 99.0%. Fusion genes were detected by F-qRT-PCR in 97.3% of patients with CML, followed by 69.4% with AML, 33.3% with acute lymphoblastic leukemia (ALL), 9.1% with myelodysplastic syndromes (MDS), and 0% with chronic lymphocytic leukemia (CLL).

**Conclusions:**

We describe the use of a F-qRT-PCR-based multi-fusion gene screening method as an efficient one-step diagnostic procedure as an effective alternative to lengthy conventional diagnostic procedures requiring both cytogenetic analysis followed by targeted quantitative reverse transcription (qRT-PCR) methods, thus allowing timely patient management.

## Background

Fusion genes are genetic chromosomal aberrations formed by the juxtaposition of two disparate gene loci through chromosomal translocation, interstitial deletion, or inversion and is most frequently detected through cytogenetic abnormalities. Although hundreds of fusion genes have been associated with leukemia, only a small portion of these fusion events are consistently recurrent and clinically characterized to the extent where they can be diagnosed and treated effectively in leukemia patients [1–3]. The prevalence of characterized gene fusions with clinical implications varies amongst differing hematological malignancies. Approximately 25-50% of acute lymphoblastic leukemia (ALL) or acute myeloid leukemia (AML) cases comprise at least one fusion gene [2, 4], while over 90% chronic myeloid leukemia (CML) cases comprise *BCR-ABL1* gene fusions [5]. Importantly, the detection of certain fusion gene events in certain hematological malignancies can act as diagnostic indicators in the selection of an effective targeted therapeutic regimen [1–3]. For example, prevalent detection of *BCR-ABL1* fusions in CML cases may be useful in prescribing tyrosine kinase inhibitor (TKI) treatment regimens such as imatinib [6], while detection of *PML-RARa* fusions may require treatments designed to treat the M3 AML subtype, acute promyelocytic leukemia (APL) [7]. In other instances, detection of fusion genes can classify malignancies into prognostic subgroups where diagnostic outcomes were not associated with targeted therapies [2, 4]. Moreover, a patient may concurrently comprise multiple oncogenic fusion genes, thereby requiring comprehensive treatment strategies targeting multiple tumorigenic components.

Currently, the detection of specific gene fusion events requires prior characterization by cytogenetic analysis to narrow possible fusion gene events to be determined by secondary screening procedures. However, cytogenetic analysis is time-consuming and may take up to 2 weeks, thus preventing immediate patient management. Further, cytogenetic analysis requires extensive training and exceptional expertise, leaving room for clinical errors that may hinder rapid and accurate diagnosis and proper treatment. Thus, there is a need to develop screening methods to bypass time constraints and clinical errors that may be associated with, cytogenetic analysis. In this study, we describe effective use of a fluorescent real-time quantitative reverse transcription PCR (F-qRT-PCR)-based multi-fusion gene screening method with the capacity to accurately screen 22 common fusion genes concurrently without prior characterization of chromosome translocation phenotypes by cytogenetic analysis. Moreover, this method can also accurately detect co-existing fusion genes, while conventional qRT-PCR methods commonly detect single gene fusions. Our method described here would greatly improve diagnostic efficiency and accuracy in leukemia, allowing immediate therapeutic management to achieve optimal outcome.

## Methods

### Patients

A total of 345 patients from Henan Cancer Hospital participated in this study. These patients were diagnosed with AML (108 patients), ALL (60), Chronic lymphocytic leukemia (CLL) (15), CML (41), Myelodysplastic Syndromes MDS (33), or other non-leukemia hematological diseases including lymphoma and cytopenia (88) from April 2014 to September 2015. Bone marrow and peripheral blood samples were collected from all participating patients for disease diagnosis and experiments proposed in this study, and written informed consent was obtained from all participants in this study. This study was approved by the Institutional Ethics Committee of Henan Cancer Hospital (2103ys34). The group of 108 patients with AML were used an “evaluation cohort” to evaluate the efficiency of the F-qRT-PCR method (described below), while the remaining patients were used as the “validation cohort.” The clinicopathological features of the 108 AML patients from the evaluation cohort are listed in Table 1.

**Table 1:** Clinical characteristics of 108 patients with AML

Variable	Cases	Percent
Age		
0-12	9	8.3%
13-19	10	9.3%
20-29	17	15.7%
30-39	22	20.4%
40-49	25	23.1%
50-59	15	13.9%
60-75	10	9.3%
Sex		
Male	62	57.4%
Female	46	42.6%
Subtypes		
M1	4	3.7%
M2	21	19.4%
M3	46	42.6%
M4	3	2.8%
M5	25	23.1%
M6	2	1.9%
Undecided	7	6.5%
Cytogenetics		
Normal	28	25.9%
abnormal	80	74.1%

### Detection of 22 fusion genes using fluorescent real-time RT-PCR

F-qRT-PCR was performed to detect 22 fusion genes (Table 2) using a Leukemia Related Fusion Gene Detection Kit (Yuntai Bio-Pharmaceutical Co., Ltd., Suzhou, China) according to the manufacturer’s instruction. 1–2 mg bone marrow aspiration samples were suspended in 200 μl saline and mixed with 500 μl Trizol (1:3) for total RNA extraction using a RNeasy Mini kit system (Qiagen, CA, USA). RNA quality was examined using the Human Tissue RNA Quality Control Kit (Yuntai Bio-Pharmaceutical Co., Ltd.), a supporting RNA quality control system for use in conjunction with the F-qRT-PCR kit. 1–2 μg total RNA was then reverse-transcribed into cDNA in a 20 μl reaction volume, which was subsequently diluted into a final volume of 40 μl. Five μls of the diluted cDNA was used for each PCR reaction, and mixed with 13 μls of PCR taq-polymerase master mixture and 7 μl primers. H_2_O and plasmid encoding leukemia cDNA fragments were used to replace cDNA templates to serve as negative and positive controls, respectively. Three PCR reactions were simultaneously conducted in each PCR reaction tube using three fluorescently-labeled (FAM, HEX and CY5) probes. A multi-PCR reaction tube system using a set of 6 reaction tube was used to detect up to 22 fusion genes in a single assay set (Table 3). PCR amplification was performed with a protocol modified from a published study [8, 9], using initial incubation at 42 °C for 5 min and at 95 °C for 10 min followed by 40 cycles of consecutive amplification at 94 °C for 15 s and 60 °C for 1 min using a 7500 Real Time PCR System (ABI, USA). Using a plasmid DNA template, the detection limit was determined to be 1000 DNA copies per reaction, while the detection specificity for both positive and negative controls were assayed at 100%. PCR products successfully amplified from gene fusion products were subcloned into plasmid vectors (pCDNA3 or equivalent) with T7 or SP6 promoter sequencing primer sites and subjected to Sanger sequencing using a 3130 Genetic Analyzer (ABI, USA) system according to the manufacturer’s instructions to verify the sequence of the amplified fusion fragment. Diagnostic results obtained by F-qRT-PCR were compared to results obtained from cytogenetic analysis. The concordance rate was calculated as the number of samples positively diagnosed by both detection methods, plus the number of samples with negative diagnoses by both detection methods, as a fraction of the total sample number.

**Table 2:** 22 fusion genes screened using the multi-fusion gene F-qRT-PCR assay system

Fusion genes	Also known as	Frequently associated diseases
*RUNX1-RUNX1T1*	*AML1-ETO*	AML M2
*AML1-MDS1/EVI1*		AML
*AML1-MTG16*	*RUNX1–CBFA2T3*	AML, MDS
*BCR-ABL*		CML, ALL
*CBFβ-MYH11*		AML
*DEK-CAN*		AML, MDS
*E2A-HLF*		Pre-B ALL
*E2A-PBX1*		Pre-B ALL
*MLL-AF10*	*KMT2A-MLLT10*	AML M5/M4
*MLL-AF4*	*KMT2A-AFF1*	ALL, AML M4/M5
*MLL-AF6*	*KMT2A-MLLT4*	AML M4/M5, T-cell ALL
*MLL-AF9*	*KMT2A-MLLT3*	AML M5a
*MLL-ELL*	*KMT2A-ELL*	AML M4/M5
*MLL-ENL*	*KMT2A-MLLT1*	AML M4/M5, B-cell ALL
*NPM-MLF*		AML M2/4/6, MDS
*PLZF-RARα*		AML M3 (APL)
*PML-RARα*		AML M3 (APL)
*STAT5b-RARα*		AML M3 (APL)
*FIP1L1-PDGFRA*		CMML, CEL
*TEL-PDGFRB*		CMML, CEL
*SIL-TAL1*		T-cell ALL
*TEL-AML1*	*ETV6-RUNX1*	B-cell ALL

*AML* acute myeloid leukemia, *ALL* acute lymphocytic leukemia, *CML* chronic lymphocytic leukemia, *MDS* myelodysplastic syndromes, *APL* acute promyelocytic leukemia, *CMML* chronic myelomonocytic leukaemia, *CEL* chronic eosinophilic leukemia

**Table 3:** PCR reaction tube system

PCR tube	Target gene	Fluorescent Channel
PCR tube 1	*BCR-ABL*	FAM
	*SIL-TAL1*	HEX
	*E2A-HLF*	CY5
PCR tube 2	*TEL-AML1*	FAM
	*MLL-AF4*	HEX
	*E2A-PBX1*	CY5
PCR tube 3	*RUNX1-RUNX1T1*	FAM
	*MLL-AF9*	HEX
	*PML-RARα*	CY5
PCR tube 4	*MLL-AF6, −AF10, −ELL, −ENL*	HEX
	*PLZF-RARα,STAT5b-RARα*	FAM
	*NPM-MLF*	CY5
PCR tube 5	*TEL-PDGFRB*	FAM
	*FIP1L1-PDGFRA*	HEX
	*AML1-MDS1/EVI1,AML1-MTG16*	CY5
PCR tube 6	*CBFβ-MYH11*	FAM
	*DEK-CAN*	HEX
	*ABL* (reference gene)	CY5

### Cytogenetic analysis

Conventional cytogenetic analysis of bone marrow was conducted in the cytogenetic laboratory at the Affiliated Cancer Hospital of Zhengzhou University using standard protocol as described elsewhere [10]. Chromosomes were prepared using a direct preparation method or a short-term culture (24 h) of bone marrow cells and visualized with R-banding. At least 20 cells were examined where possible, although the experiments with less than 20 cells will not be excluded. The karyotype was described according to the International System for Human Cytogenetic Nomenclature (ISCN).

## Results

**Fig. 1:**
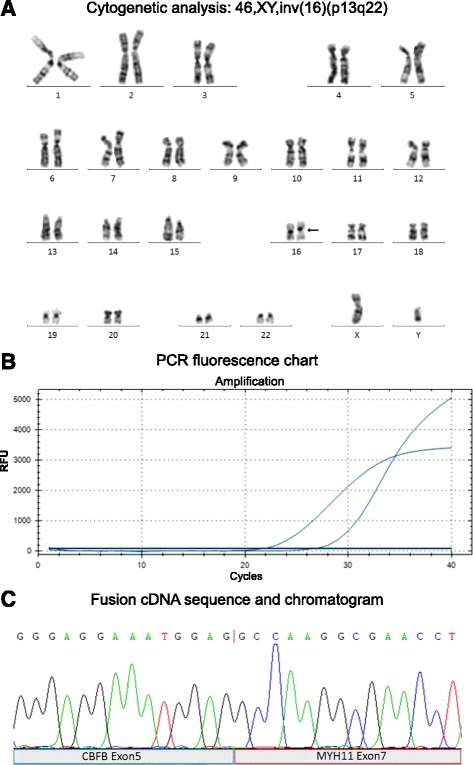
Molecular and cytogenetic screening in hematological malignancies. **a** Cytogenetic analysis of bone marrow biopsy identified with a karyotypic chromosomal rearrangement inv(16)(p13;q22) (*black arrow*). **b** PCR fluorescent chart showing amplification curve for both targeted gene fusion and housekeeping reference cDNA products. **c** DNA sequence (*top*) and chromatograph (*bottom*) of a CBFβ-MYH11 fusion transcript (cDNA). Chromosomal breakpoint is indicated by a *red line* within the DNA sequence. Contributing exons from each gene (DBFB exon 5 and MYH11 exon 7) are indicated below (*blue and red shaded labeled regions*)

We first evaluated the efficiency of the F-qRT-PCR diagnostic system using the 108 AML patients comprising the evaluative cohort. All patients were characterized for cytogenetic abnormalities to include chromosome translocations by cytogenetic analysis (Fig. 1a). F-qRT-PCR was performed on all patients in a blinded manner without prior knowledge of translocation classification obtained from cytogenetic characterization (Fig. 1b). mRNA gene fusion products successfully detected by F-qRT-PCR were purified and subjected to Sanger sequencing analysis to validate amplification of the chromosomal mRNA fusion product (Fig. 1c). Results obtained from cytogenetic and F-qRT-PCR analysis were compared to gauge diagnostic accuracy (Table 4). From the AML patient cohort, 34 patients (Cyto-) showed no chromosomal rearrangement with 28 patients assayed with normal karyotypes and 6 patients observed with abnormal cytogenetic morphologies. Chromosomal rearrangements were identified in the remaining 74 cases (Cyto+), implicating the occurrence of the following chromosomal gene fusions: *RUNX1-RUNX1T1*, *AML1-MDS/EVII/MTG16*, *FIP1L1-PDGFRA*, *PML-RARa*, *CBFb-MYH11*, *SIL-TAL1*, or *MLL-AF6/AF9/AF10/ELL/ENL*. We successfully identified all predicted gene fusion events in the 74 cases characterized with chromosomal arrangements using the F-qRT-PCR diagnostic system. In addition, we detected a *RUNX1-RUNX1T1* gene fusion by F-qRT-PCR in a Cyto- case, indicating that use of our F-qRT-PCR method may have superior sensitivity in comparison to cytogenetic analysis. Comparing F-qRT-PCR to cytogenetics, the sensitivity and specificity were 100.0 and 97.1%, respectively and the overall concordance rate between the two methods was determined to be 99.1% (Table 5).

**Table 4:** Comparison of diagnostic results obtained by cytogenetic analysis and multi-fusion gene F-qRT-PCR screening

Subtype	Cytogenetics				F-qRT-PCR			
	Cyto-	Cyto+	% (+)	Translocation	PCR-	PCR+	% (+)	Fusion gene
M1	4		0%	none	4		0%	None
AML M2	1			none	1			None
	^a^ **1**		**90.5%**	**none**		**1**	**95.2%**	***RUNX1-RUNX1T1***
		17		t(8;21)(q22;q22)		17		*RUNX1-RUNX1T1*
		1		t(16;21)(q24;q22)		1		*AML1-MDS/EVII/MTG16*
		1		del(4)(q12)		1		*FIP1L1-PDGFRA*
AML M3	3			none	3			None
		43	93.5%	t(15;17)(q22;q12)		43	93.5%	*PML-RARα*
AML M4	2			none	2			None
		1	33.3%	t(8;21)(q22;q22)		1	33.3%	*RUNX1-RUNX1T1*
AML M5	22			none	22			None
		2	12.0%	inv(16)(p13;q22)		2	12.0%	*CBFβ-MYH11*
		1		t(9;11)(p22;q23)		1		*MLL-AF9*
AML M6	1			none	1			None
		1	50.0%	del(1p)		1	50.0%	*SIL-TAL1*
Undecided		3	100.0%	inv(16)(p13;q22)		3	100.0%	*CBFβ-MYH11*
		3		t(6;11)(q26;q23)		3		*MLL-AF6/AF10/ELL/ENL*
		1		del(1p)		1		*SIL-TAL1*
subtotal	34	74	68.5%		33	75	69.4%	

^a^Bold print indicates cases with inconsistent results obtained using the two diagnostic methods

**Table 5:** Concordance rates determined between cytogenetic analysis and multi-fusion gene F-qRT-PCR screening in 108 AML patients

	PCR+	PCR-	Total	Sensitivity	Specificity	Concordance rate
Cyto+	74	0	74	100.0%		
Cyto-	1	33	34		97.1%	
Total	75	33	108			99.1%

We next expanded our study to other leukemic subtypes to include 60 ALL, 15 CLL, 41 CML and 33 MDS, or non-leukemic disorders. Combined with the aforementioned AML cases, a total of 345 patients were characterized (Table 6). Within this new cohort, cytogenetic analysis predicted chromosomal rearrangements (Cyto+) in 144 patients, where chromosome rearrangements were not detected in (Cyto-) in 201 patients. A total of 4 cases (including 1 patient described in the AML evaluation cohort) showed discrepancies between F-qRT-PCR and cytogenetic diagnostic assays. In addition to the aforementioned Cyto- patient in the AML cohort, gene fusions were detected in two additional Cyto- cases, including an ALL patient with *BCR-ABL* rearrangements and a CML patient with *AML-ETO* fusions. However, we failed to detect expression of a *BCR-ABL* fusion in a CML patient with cytogenetic 46,xy,t(9;22)(q34;q11)[1]/46,xy[9] rearrangements by F-qRT-PCR. Nonetheless, we determined the concordance rate between F-qRT-PCR and cytogenetic analysis methods to be 98.8%, as determined by the sum of PCR-/Cyto- (198) and PCR+/Cyto + (143) concordant cases as a ratio of the total number of cases (345).

**Table 6:** Cases with fusion genes detected by FQ- RT-PCR in 34 leukemia patients

Gene Fusions	ALL	AML	CLL	CML	MDS	Other Leukemias	Others	Subtotal
*RUNX1-RUNX1T1*		19 (18)^a^		1 (0)^a^	1	21 (19)	1	22 (20)
*AML1-MDS/EVII/MTG16*		1				1		1
*BCR-ABL*	5 (4)^a^			37 (38)^b^		42 (42)		42 (42)
*CBFβ-MYH11*		5				5		5
*E2A-PBX1*	7					7		7
*FIP1L1-PDGFRA*		1				1	1	2
*MLL-AF4*	3					3	1	4
*MLL-AF6/AF10/ELL/ENL*	1	3				4	3	7
*MLL-AF9*	1	1				2		2
*NPM-MLF1*					1	1		1
*PML-RARα*		43		1		44		44
*SIL-TAL1*		2		1		3	2	5
*TEL-AML1*	3					3		3
*TEL-PDGFRB*					1	1		1
Subtotal	20 (19)	75 (74)	0	40 (40)	3	138 (136)	8	146 (144)
No fusion	40 (41)	33 (34)	15	0 (1) + 1 (0)	30	119 (121)	80	199 (201)
Total	60	108	15	41	33	257	88	345
% (fusion/total) by PCR	33.3%	69.4%	0.0%	97.6%	9.1%	53.7 (52.9)%	9.1%	42.3 (41.7)%

Numbers in parentheses are predicted by cytogenetic analysis

^a^cytogenetic-negative, PCR-positive

^b^cytogenetic-positive PCR-negative cases

We detected gene fusions in 138 out of 257 leukemia cases by F-qRT-PCR, accounting for 53.7% of the patients assayed. Gene fusions were observed most abundantly in CML patients at a frequency of 97.6% patients assayed, followed by AML (69.4%), ALL (33.3%), and MDS (9.1%). No fusion genes were detected in any of the 15 CLL patients characterized in this study. Together, these results indicate that leukemia rearrangements can be rapidly and accurately diagnosed by the F-qRT-PCR method described here, which may facilitate timely application of proper treatment strategies to optimize patient outcome.

## Discussion

Accurate and efficient diagnosis in leukemia using reliable detection methods is essential in prescribing effective tumor treatment strategies and relevant targeted therapeutic regimens. Although the characterization of chromosomal rearrangements and gene fusion events is crucial to successful diagnosis and treatment in leukemia, clinical diagnosis may be complicated by factors such as limitations in sample availability, error-prone, lengthy analytic procedures and costly procedural expenses. In this study, we describe the use of a F-qRT-PCR-based multi-fusion gene screening method to rapidly and accurately characterize chromosomal gene fusions in leukemia patients. The efficiency of this method is exemplified by a rapid turnaround time, and presents a cost-effective diagnostic alternative to conventional methods that require multiple procedural assays, such as cytogenetic karyotyping and FISH, followed by qRT-PCR to characterize a single oncogenic chromosomal aberration. Moreover, this method can detect a comprehensive set of 22 frequently recurrent fusion genes in a single experiment with superior accuracy and rapid turnaround times.

Conventional diagnostic tests in leukemic patients include comprehensive blood analysis, peripheral blood smear analysis, bone marrow tests, immunophenotyping (CD antigen expression) and intensive chromosomal characterization assays (cytogenetics, FISH and RT-PCR). These labor-intensive measures, however, are necessary to accurately determine oncogenic chromosomal gene rearrangements which give indication to an optimal treatment strategy. Specific cytogenetic abnormalities in over 25-50% of patients with ALL or AML [2, 4] and over 90% of patients with CML [5] have been previously described, giving further indication that specific leukemic gene rearrangements require accurate diagnosis to determine a proper treatment regimen. For decades, characterization of leukemic subtypes involved initial analysis by cytogenetic examination to pre-determine chromosomal translocation or rearrangements, which would then be subsequently defined by a combination of analysis by FISH, immunohistochemistry, RT-PCR and Sanger sequencing. Since both FISH and conventional RT-PCR analytic methods are limited by their ability to detect one particular chromosomal translocation, inversion, or deletion event, prior cytogenetic analysis was required to predetermine which particular chromosomal aberrations required further characterization by FISH or RT-PCR. As cytogenetic analysis may take up to 2 weeks, F-qRT-PCR screening procedures as described here may bypass lengthy cytogenetic pre-screening requirements, with the capacity to identify 22 fusion genes commonly associated with leukemia, which can expedite patient management strategies to optimize patient outcome. It should be noted that cytogenetic analysis is still of importance in characterizing other types of chromosomal abnormalities, including aneuploidy, in hypodiploid or hyperdiploid ALL and other leukemic subtypes.

FISH is frequently used to detect and confirm predefined gene fusion events in leukemia. The standard turnaround time for FISH analysis is 3 days in our hospital, while it can be done within 24-h by rapid hybridization. Similar to qRT-PCR, FISH can also be used to document the disease burden and detect residual disease [11, 12], while qRT-PCR may exhibit better efficiency and sensitivity [13]. However, successful application of FISH analysis is dependent on the predetermination of chromosomal translocation events as defined by cytogenetic assay and other clinical assay methods in order to select and use appropriate DNA probes to detect specific chromosomal rearrangements [14]. Although FISH panels have been employed to detect more than one type of chromosomal rearrangement, this procedure is often costly, labor-intensive and technically difficult which may prevent a universal capacity to produce reliable and affordable diagnoses using this method. In contrast, RT-PCR-based methods require less training and experience, and application of RT-PCR analyses have become standard in clinical diagnostic procedures. As shown in Table 7, the cost and turnaround time in our hospital are higher for FISH or FISH panels than for single qRT-PCR or F-qRT-PCR, respectively. Moreover, our F-qRT-PCR covers a much broader spectrum of diseases than individual FISH panels, which are usually designed to cover one particular subtype of leukemia disease with very limited types of chromosomal rearrangements. It would require multiple FISH panels to cover the same number of fusion genes covered by our F-qRT-PCR method. Moreover, qRT-PCR has achieved greater sensitivity with enhanced flexibility in sample quantity and quality [15]. In the study described above, we used both target-specific primers and fluorescent probes to enhance signal specificity and quantitative fidelity. To overcome the lack of multiplexing capability [16, 17], we used a multi-PCR reaction tube system by simultaneously amplifying three PCR reaction sets using three fluorescently-labeled (FAM, HEX and CY5) probes. Using this method, we were able to detect up to 22 fusion genes in a single assay set.

**Table 7:** Comparison of different diagnostic methods in Henan Cancer Hospital

Diagnostic methods	Required day (s)	Price ($)
Cytogenetic analysis	14	$132.31
FISH for BCR/ABL	3	$165.63
qRT-PCR for single fusion gene	1	$55.38
FISH panel for MDS	3	$406.54
F-qRT-PCR panel for 22 common leukemic fusions	1	$253.85

This advanced screening method allowed us to detect gene fusions in 53.7% of leukemia patients. In agreement with previous reports, we detected fusion genes in 69.4% patients with AML, and 33.3% with ALL [2, 4]. Additionally, we detected fusion genes in 97.6% or BCR-ABL in 90.2% of all CML patients characterized, which is also in good agreement to CML-associated gene fusions reported previously [3]. In this study, 4 cases revealed inconsistent outcomes using cytogenetic and F-qRT-PCR methods. Although three of these cases failed to reveal abnormalities by cytogenetic analysis, gene fusions were identified in these individuals by F-qRT-PCR, which was confirmed by direct sequencing of the resulting PCR products. These results were verified immediately by subsequent duplicating assays. The failure to detect genetic abnormalities by cytogenetic analysis is likely due to but not limited to one or more of the following reasons: (1) cytogenetic analysis is less sensitive than F-qRT-PCR; (2) The chromosomal rearrangements occurred in an extremely low percentage of tumor cells due to the high genetic heterogeneity in these cases; (3) Technical errors may occur by chance in certain cases, especially since this method demands tremendous skill and experience; (4) The quality of the samples was poor due to poor preservation, due to various reasons; (5) cytogenetic analytic procedures were inconsistent, perhaps due to sample limitations and technically difficult repeatability, which may be exacerbated by the labor-intensive and costly nature of the procedure. Chromosomal abnormalities were revealed in one case by cytogenetic analysis, which failed to reveal any gene fusions by F-qRT-PCR analysis. This incidence is likely an extremely rare event as leukemia is extremely heterogeneous with only 1 of 10 cells harboring a t(9,22) translocation have been detected by a subsequent cytogenetic assay.

In conclusion, we describe an advanced F-qRT-PCR-based multi-fusion gene screening method using target-specific primers and fluorescent-labeled hybridization probes to detect 22 common leukemic fusion genes in a single assay system. This method is extremely efficient with reduced turnaround times, and shows enhanced sensitivity and specificity for a wide target range. Accurate detection and characterization of gene fusions using this method can be comprehensively accomplished within 1 to 2 days without prior chromosomal characterization by cytogenetic analysis. The methods described in this study may potentially enhance the efficiency and accuracy of diagnosis of leukemic patients, which may greatly affect current procedural costs and therapeutic management.

## Conclusions

Use of a F-qRT-PCR multi-gene fusion detection system described here can rapidly and accurately detect and characterize 22 gene fusion events commonly found in leukemia with comparable accuracy to conventional cytogenetic methods. As conventional diagnostic methods require pre-characterization of chromosomal abnormalities by cytogenetic analysis, this system bypasses the need for cytogenetic pre-characterization, thus expediting diagnosis and patient treatment management in leukemia subtypes characterized with gene fusions.

## Abbreviations

ALL: Acute lymphoblastic leukemia
AML: Acute myeloid leukemia
APL: Acute promyelocytic leukemia
CLL: Chronic lymphocytic leukemia
CLL: Chronic lymphocytic leukemia
FISH: Fluorescent in situ hybridization
F-qRT-PCR: Fluorescent real-time RT-PCR
MDS: Myelodysplastic syndromes
